# Supplementary material for: Association between arterial stiffness and Loa loa microfilaremia in a rural area of the Republic of Congo: A population-based cross-sectional study (the MorLo project)
Source: PLoS Negl Trop Dis. 2024 Jan 19;18(1):e0011915. doi: 10.1371/journal.pntd.0011915 (PMC10830006; doi:10.1371/journal.pntd.0011915)
Supplement: S1 Table — Abbreviations: N, number of subjects in the category; MFD, microfilarial density. * Values calculated from individuals with no hypertension (defined as SBP <140 mmHg and DPB <90 mmHg), non-smokers, non-obese/in overweight and with no L. loa microfilaremia. ** Values calculated from individuals with no hypertension (defined as DPB <90 mmHg), non-smokers, non-obese/in overweight and with no L. loa microfilaremia. (DOCX) [file pntd.0011915.s001.docx]

**S1 Table.** Pulse Wave Velocity measurements

|  | **PWV in m/s**  mean ± SD  median [10^th^ – 90^th^ percentile]) | | | | | |  |
| --- | --- | --- | --- | --- | --- | --- | --- |
| **Age category (years)** | **<30** | **30–39** | **40–49** | **50–59** | **60–69** | **≥70** |  |
| **Reference Values from the Arterial Stiffness' Collaboration**^23^ | | | | | | |  |
|  | 5.0 ± 0.6  4.9 [4.2–5.7] | 5.2 ±1.1  5.1 [4.2–6.4] | 5.8 ±1.0  5.5 [4.7–6.9] | 6.6 ± 1.5  6.5 [5.0–8.0] | 8.2 ± 1.9  7.8 [6.3–10.5] | 8.7 ± 2.2  8.5 [6.4–11.7] |  |
| **Reference values #1 for our population study ^*^** | | | | | | | |
| Males | N = 22  5.6 ± 0.5  5.7 [5.1–6.1] | N = 18  6.0 ± 0.7  5.9 [5.4–7.2] | N = 29  6.7 ± 0.9  6.5 [5.7–8.2] | N = 22  7.6 ± 1.2  7.7 [6.1–9.1] | N = 9  7.4 ± 2.3  6.6 [5.6–12.8] | N = 8  9.5 ± 3.2  8.5 [6.4–15.0] |  |
| Females | N = 12  5.4 ± 0.4  5.2 [4.8–6.0] | N = 13  6.1 ± 0.8  6.4 [5.2–6.9] | N = 24  6.5 ± 1.3  6.2 [5.4–7.7] | N = 21  7.5 ± 1.9  7.1 [5.9–10.4] | N = 11  8.8 ± 2.3  8.5 [6.7–11.3] | N = 6  10.4 ± 1.3  10.2 [9.2–12.5] |  |
| **Reference values #2 for our population study ^**^** | | | | | | |  |
| Males | N = 22  5.6 ± 0.5  5.7 [5.1–6.1] | N = 21  6.0 ± 0.7  5.9 [5.4–7.2] | N = 34  6.7 ± 0.8  6.6 [5.8–8.1] | N = 26  7.6 ± 1.3  7.6 [6.1–9.1] | N = 15  8.6 ± 2.9  7.7 [5.7–12.9] | N = 10  9.8 ± 2.9  9.6 [6.5–13.7] |  |
| Females | N = 12  5.4 ± 0.6  5.2 [4.8–6.0] | N = 13  6.1 ± 0.8  6.4 [5.2–6.9] | N = 26  6.5 ± 1.3  6.2 [5.4–7.7] | N = 32  7.8 ± 1.7  7.2 [6.0–10.1] | N = 22  9.8 ± 3.5  8.7 [7.3–12.0] | N = 10  10.8 ± 3.4  10.0 [7.9–16.1] |  |
| ***Loa* microfilaremia status** | | | | | | | |
| Negative | N = 67  5.7 ± 0.8 5.6 [4.7–6.8] | N = 84  6.2 ± 0.9  6.2 [5.2–7.3] | N = 140  7.0 ± 1.4  6.7 [5.5–8.8] | N = 158  7.8 ± 1.8  7.4 [5.9–10.1] | N = 124  10.7 ± 5.0  9.5 [6.8–16.8] | N = 69  11.4 ± 4.8  11.0 [7.0–16.1] |  |
| Positive | N = 28  6.1 ± 0.8  6.1 [5.2–6.9] | N = 54  6.2 ± 1.0  6.0 [5.1–7.4] | N = 62  6.8 ± 1.2  6.8 [5.4–8.3] | N = 90  8.3 ± 2.1  7.8 [6.3–10.9] | N = 69  9.3 ± 3.1  8.6 [6.0–12.9] | N = 37  12.8 ± 5.9  11.9 [7.3–21.5] |  |
| ***Loa* MFD categories (n, %)** | | | | | | | |
| 1–499 mfs/mL | N = 7  6.7 ± 1.2  6.4 [5.3–8.9] | N = 13  5.9 ± 1.0  5.7 [5.0–6.9] | N = 15  6.9 ± 1.2  6.6 [5.7–8.7] | N = 20  8.4 ± 1.8  8.5 [5.8–11.0] | N = 22  9.3 ± 2.2  8.8 [7.5–12.9] | N = 14  10.9 ± 4.5  9.4 [7.2–14.9] |  |
| 500–2,499 mfs/mL | N = 6  5.7 ± 0.6  5.7 [4.7–6.5] | N = 16  6.1 ± 0.7  6.0 [5.3–7.4] | N = 12  6.7 ± 0.8  6.9 [5.8–7.4] | N = 26  8.4 ± 2.3  7.8 [6.3–11.6] | N = 14  8.3 ± 2.8  8.1 [5.4–11.2] | N = 6  12.8 ± 3.7  12.5 [8.3–18.7] |  |
| 2,500–9,999 mfs/mL | N = 8  5.9 ± 0.6  6.0 [4.9–6.6] | N = 15  6.2 ± 0.9  6.0 [5.1–7.3] | N = 16  6.6 ± 1.2  6.4 [4.7–8.0] | N = 22  7.7 ± 1.2  7.5 [6.5–9.3] | N = 21  9.9 ± 4.1  8.9 [5.7–12.9] | N = 11  15.4 ± 7.9  12.6 [11.0–21.5] |  |
| ≥10,000 mfs/mL | N = 7  6.3 ± 0.5  6.3 [5.4–6.9] | N = 10  7.0 ± 1.1  7.0 [5.6–8.7] | N = 19  7.1 ± 1.3  6.9 [5.3–9.2] | N = 22  8.8 ± 2.7  8.0 [6.6–10.7] | N = 12  9.4 ± 2.9  8.9 [6.3–12.0] | N = 6  12.4 ± 5.7  11.0 [7.3–22.8] |  |

Abbreviations: N, number of subjects in the category; MFD, microfilarial density.

* Values calculated from individuals with no hypertension (defined as SBP <140 mmHg and DPB <90 mmHg), non-smokers, non-obese/in overweight and with no *L. loa* microfilaremia.

** Values calculated from individuals with no hypertension (defined as DPB <90 mmHg), non-smokers, non-obese/in overweight and with no *L. loa* microfilaremia.
