# Supplementary material for: Association between arterial stiffness and Loa loa microfilaremia in a rural area of the Republic of Congo: A population-based cross-sectional study (the MorLo project)
Source: PLoS Negl Trop Dis. 2024 Jan 19;18(1):e0011915. doi: 10.1371/journal.pntd.0011915 (PMC10830006; doi:10.1371/journal.pntd.0011915)
Supplement: S2 Table — Abbreviations: PWV, pulse wave velocity; N., number; Hb1AC, glycated hemoglobin; SD, standard deviation; IQR, interquartile range; HDL, high density lipoprotein; LDL, low density lipoprotein; NA, not applicable. * An individual is defined as out of range if its PWV is higher than the 90th percentile of the population considered healthy in the same age category (see S1 Table–References values #1). ** Threshold at which the measurement is considered out of range: Hb1Ac >7%; Total cholesterol >5 mmol/L; Triglycerides >1.7 mmol/L; HDL <1.0 mmol/L; LDL >3.5 mmol/L; for lipid panel, measurement is considered out of range if one of the lipids is out of range. *** Chi-2 test for categorical variables with all effectives > 5 or fisher’s exact test. (DOCX) [file pntd.0011915.s002.docx]

**S2 Table**. Lipid profile and glycated hemoglobin according to the PWV status.

|  | **Total** | **PWV** | |  |
| --- | --- | --- | --- | --- |
|  |  | Normal | Out of range * | p value *^***^* |
| N. subjects (n, %) | 982 | 790 (80.5%) | 192 (19.5%) |  |
| Hb1Ac (%) |  |  |  |  |
| N. of measurements (n, %) | 238 (24.2%) | 110 (13.9%) | 128 (66.6%) |  |
| Out of range measurements (n, %) | 1 (0.4%) | 1 (0.9%) | 0 (0%) | NA |
| Mean ± SD | 5.0 ± 0.5 | 5.1 ± 0.5 | 5.0 ± 0.4 |  |
| Median [IQR] | 5.0 [4.8–5.3] | 5.1 [4.8–5.3] | 5.0 [4.8–5.3] |  |
| Lipid panel |  |  |  |  |
| N. of measurements (n, %) | 230 (23.4%) | 107 (13.5%) | 123 (64.1%) |  |
| Out of range measurements (n, %) ^**^ | 82 (35.6%) | 42 (51.2%) | 40 (48.8%) | .289 |
| Total cholesterol (mmol/L) |  |  |  |  |
| Out of range measurements (n, %) ^**^ | 29 (12.4%) | 13 (12.1%) | 16 (13.0%) | .668 |
| Mean ± SD | 3.8 ± 1.1 | 3.9 ± 1.2 | 3.7 ± 1.0 |  |
| Median [IQR] | 3.7 [3.0–4.4] | 3.7 [3.1–4.4] | 3.7 [3.0–4.5] |  |
| Triglycerides (mmol/L) |  |  |  |  |
| Out of range measurements (n, %) ^**^ | 17 (7.3%) | 7 (6.5%) | 10 (8.1%) | .671 |
| Mean ± SD | 1.1 ± 0.4 | 1.1 ± 0.4 | 1.1 ± 0.5 |  |
| Median [IQR] | 1.0 [0.7–1.3] | 1.0 [0.7–1.3] | 1.0 [0.7–1.3] |  |
| HDL (mmol/L) |  |  |  |  |
| Out of range measurements (n, %) ^**^ | 53 (22.7%) | 27 (25.2%) | 26 (21.1%) | .396 |
| Mean ± SD | 1.3 ± 0.4 | 1.3 ± 0.4 | 1.3 ± 0.4 |  |
| Median [IQR] | 1.3 [1.0–1.5] | 1.3 [1.0–1.6] | 1.2 [1.0–1.5] |  |
| LDL (mmol/L) |  |  |  |  |
| Out of range measurements (n, %) ^**^ | 13 (5.6%) | 6 (5.6%) | 7 (5.7%) | .925 |
| Mean ± SD | 2.0 ± 0.8 | 2.0 ± 0.9 | 2.0 ± 0.7 |  |
| Median [IQR] | 1.9 [1.5–2.5] | 1.9 [1.4–2.5] | 1.9 [1.5–2.5] |  |

**Abbreviations:** PWV, pulse wave velocity; N., number; Hb1AC, glycated hemoglobin; SD, standard deviation; IQR, interquartile range; HDL, high density lipoprotein; LDL, low density lipoprotein; NA, not applicable.

^*^ An individual is defined as out of range if its PWV is higher than the 90^th^ percentile of the population considered healthy in the same age category (see Table S1 – References values #1).

^**^ Threshold at which the measurement is considered out of range: Hb1Ac >7%; Total cholesterol >5 mmol/L; Triglycerides >1.7 mmol/L; HDL <1.0 mmol/L; LDL >3.5 mmol/L; for lipid panel, measurement is considered out of range if one of the lipids is out of range.

^***^ Chi-2 test for categorical variables with all effectives > 5 or fisher’s exact test
