# Supplementary material for: Association between arterial stiffness and Loa loa microfilaremia in a rural area of the Republic of Congo: A population-based cross-sectional study (the MorLo project)
Source: PLoS Negl Trop Dis. 2024 Jan 19;18(1):e0011915. doi: 10.1371/journal.pntd.0011915 (PMC10830006; doi:10.1371/journal.pntd.0011915)
Supplement: S3 Table — Abbreviations: PAD, peripherical arterial disease; N., number; Hb1AC, glycated hemoglobin; SD, standard deviation; IQR, interquartile range; HDL, high density lipoprotein; LDL, low density lipoprotein; NA: not applicable. * Threshold at which the measurement is considered out of range: Hb1Ac >7%; Total cholesterol >5 mmol/L; Triglycerides >1.7 mmol/L; HDL <1.0 mmol/L; LDL >3.5 mmol/L; for lipid panel, measurement is considered out of range if one of the lipids is out of range. ** Chi-2 test for categorical variables with all effectives > 5 or fisher’s exact test. (DOCX) [file pntd.0011915.s003.docx]

**S3 Table**. Lipid profile and glycated hemoglobin according to the PAD.

|  | **Total** | **PAD** | |  |
| --- | --- | --- | --- | --- |
|  |  | Absence | Presence | p value *^**^* |
| N. subjects (n, %) | 976 | 839 (86.0%) | 137 (14.0%) |  |
| Hb1Ac (%) |  |  |  |  |
| N. of measurements (n, %) | 236 (24.2%) | 206 (24.5%) | 30 (21.9%) |  |
| Out of range measurements (n, %) ^*^ | 1 (0.4%) | 1 (0.5%) | 0 (0%) | NA |
| Mean ± SD | 5.0 ± 0.5 | 5.1 ± 0.5 | 5.0 ± 0.4 |  |
| Median [IQR] | 5.0 [4.8–5.3] | 5.1 [4.8–5.3] | 5.0 [4.8–5.3] |  |
| Lipid panel |  |  |  |  |
| N. of measurements (n, %) | 231 (23.7%) | 202 (24.1%) | 29 (21.2%) |  |
| Out of range measurements (n, %) ^*^ | 82 (35.5%) | 71 (35.1%) | 11 (37.9%) | .769 |
| Total cholesterol (mmol/L) |  |  |  |  |
| Out of range measurements (n, %) ^*^ | 29 (12.5%) | 24 (11.9%) | 5 (17.2%) | .379 |
| Mean ± SD | 3.8 ± 1.1 | 3.8 ± 1.1 | 3.9 ± 1.0 |  |
| Median [IQR] | 3.7 [3.0–4.4] | 3.7 [3.0–4.5] | 3.9 [3.1–4.2] |  |
| Triglycerides (mmol/L) |  |  |  |  |
| Out of range measurements (n, %) ^*^ | 17 (7.3%) | 17 (8.4%) | 0 (0%) | NA |
| Mean ± SD | 1.1 ± 0.4 | 1.0 ± 0.4 | 0.9 ± 0.4 |  |
| Median [IQR] | 1.0 [0.7–1.3] | 1.0 [0.8–1.3] | 0.8 [1.6–1.1] |  |
| HDL (mmol/L) |  |  |  |  |
| Out of range measurements (n, %) ^*^ | 53 (22.9%) | 46 (22.8%) | 7 (24.1%) | .870 |
| Mean ± SD | 1.3 ± 0.4 | 1.3 ± 0.4 | 1.4 ± 0.5 |  |
| Median [IQR] | 1.3 [1.0–1.5] | 1.3 [1.0–1.5] | 1.2 [1.0–1.5] |  |
| LDL (mmol/L) |  |  |  |  |
| Out of range measurements (n, %) ^*^ | 13 (5.6%) | 11 (5.4%) | 2 (6.7%) | .670 |
| Mean ± SD | 2.0 ± 0.8 | 2.0 ± 0.7 | 2.1 ± 0.8 |  |
| Median [IQR] | 1.9 [1.5–2.5] | 1.9 [1.5–2.5] | 2.1 [1.5–2.4] |  |

**Abbreviations:** PAD, peripherical arterial disease; N., number; Hb1AC, glycated hemoglobin; SD, standard deviation; IQR, interquartile range; HDL, high density lipoprotein; LDL, low density lipoprotein; NA: not applicable

^*^ Threshold at which the measurement is considered out of range: Hb1Ac >7%; Total cholesterol >5 mmol/L; Triglycerides >1.7 mmol/L; HDL <1.0 mmol/L; LDL >3.5 mmol/L; for lipid panel, measurement is considered out of range if one of the lipids is out of range

^**^ Chi-2 test for categorical variables with all effectives > 5 or fisher’s exact test
