# Supplementary material for: Association between arterial stiffness and Loa loa microfilaremia in a rural area of the Republic of Congo: A population-based cross-sectional study (the MorLo project)
Source: PLoS Negl Trop Dis. 2024 Jan 19;18(1):e0011915. doi: 10.1371/journal.pntd.0011915 (PMC10830006; doi:10.1371/journal.pntd.0011915)
Supplement: S4 Table — 1 Fisher’s exact test. 2 Cramér’s V. 3 Cuzick’s test. * For lipid panel, an individual is considered out of range if at least one of the lipids is out of range (see S2 and S3 Tables). (DOCX) [file pntd.0011915.s004.docx]

**S4 Table**. Analysis of correlations between *Loa* *loa* microfilarial status and densities, lipid profile, glycated hemoglobin and co-infections.

|  | ***Loa loa* microfilarial status** | | | | ***Loa loa* microfilarial densities (mfs/mL)** | | | | | |
| --- | --- | --- | --- | --- | --- | --- | --- | --- | --- | --- |
|  | Positive | Negative | p value*^1^* | *V^2^* | 1–4,999 | 5,000–14,999 | ≥ 15,000 | p value*^1^* | *V^2^* | *P^3^* |
| Hb1AC |  |  | .524 | .010 |  |  |  | .803 | .063 | .359 |
| ≤ 5.5 % | 77 (36.0%) | 137 (64.0%) |  |  | 48 (22.4%) | 18 (8.4%) | 11 (5.1%) |  |  |  |
| > 5.5 % | 9 (37.5%) | 15 (62.5%) |  |  | 6 (25.0%) | 1 (4.2%) | 2 (8.3%) |  |  |  |
| Lipid panel |  |  | .152 | .102 |  |  |  | .161 | .149 | .319 |
| Normal | 48 (32.4%) | 100 (67.6%) |  |  | 27 (18.2%) | 14 (9.5%) | 7 (4.7%) |  |  |  |
| Out of range**^*^** | 35 (42.7%) | 47 (57.3%) |  |  | 24 (29.3%) | 5 (6.1%) | 6 (7.3%) |  |  |  |
| *Ascaris* *lumbricoides* |  |  |  |  |  |  |  |  |  |  |
| Presence | 130 (39.2%) | 202 (60.8%) | .096 | .061 | 76 (22.9%) | 40 (12.1%) | 14 (4.2%) | .011 | .012 | .056 |
| Absence | 146 (33.3%) | 293 (66.7%) |  |  | 100 (22.8%) | 24 (5.5%) | 22 (5.0%) |  |  |  |
| *Trichuris trichiura* |  |  |  |  |  |  |  |  |  |  |
| Presence | 87 (42.0%) | 120 (33.6%) | .034 | .078 | 49 (23.7%) | 30 (14.5%) | 8 (3.9%) | .002 | .141 | .017 |
| Absence | 190 (33.6%) | 376 (66.4%) |  |  | 128 (22.6%) | 34 (6.0%) | 28 (4.9%) |  |  |  |
| Malaria |  |  |  |  |  |  |  |  |  |  |
| Presence | 8 (50.0%) | 8 (50.0%) | .195 | .042 | 2 (12.5%) | 5 (31.3%) | 1 (6.2%) | N/A | .110 | .077 |
| Absence | 331 (34.3%) | 635 (65.7%) |  |  | 214 (22.1%) | 76 (7.8%) | 41 (4.2%) |  |  |  |

^1^ Fisher's exact test

^2^ Cramér's V

^3^ Cuzick’s test

* For lipid panel, an individual is considered out of range if at least one of the lipids is out of range (see S2 and S3 Tables)
