# Supplementary material for: Association between arterial stiffness and Loa loa microfilaremia in a rural area of the Republic of Congo: A population-based cross-sectional study (the MorLo project)
Source: PLoS Negl Trop Dis. 2024 Jan 19;18(1):e0011915. doi: 10.1371/journal.pntd.0011915 (PMC10830006; doi:10.1371/journal.pntd.0011915)
Supplement: S5 Table — Abbreviations: PWV, pulse wave velocity; N, number; SD, Standard deviation; MFD, microfilarial density; IQR, interquartile range; N/A, not applicable. * Chi-2 for categorical variable and Kruskal-Wallis rank test for continuous variables. ** An individual is defined as out of range if his/her PWV is higher than the 90th percentile of the population considered healthy in the same age category (see S1 Table 1 –References values #2). (DOCX) [file pntd.0011915.s005.docx]

**S5 Table**. Distribution of the main characteristics according to Pulse Wave Velocity status (using References values #2).

|  | **Total** | **PWV** | | **p value ***** |
| --- | --- | --- | --- | --- |
|  |  | Normal | Out of range ** |  |
| N. subjects (n, %) | 982 | 801 (81.6%) | 181 (18.4%) |  |
| Sex-ratio (M/F) | 1.67 | 1.60 | 2.07 | 0.141 |
| Age in years (mean ± SD) | 50.9 ± 14.8 | 51.2 ± 14.4 | 49.2 ± 16.3 | 0.164 |
| Average blood pressure in mmHg (mean ± SD) | 95.3 ± 16.6 | 94.1 ± 15.6 | 100.6 ± 19.3 | <0.001 |
| Pulse rate in bpm (mean ± SD) | 64.1 ± 12.3 | 63.5 ± 12.1 | 66.8 ± 12.9 | 0.002 |
| Body mass index (mean ± SD) | 21.0 ± 3.2 | 21.0 ± 3.2 | 21.2 ± 3.1 | 0.532 |
| Smoking (n, %) | 181 (18.6%) | 149 (18.2%) | 32 (17.9%) | 0.794 |
| Creatininemia in µmol/L (mean ± SD) | 71.2 ± 19.3 | 70.7 ± 19.0 | 74.5 ± 19.2 | 0.011 |
| Malaria presence (n, %) | 16 (1.6%) | 14 (1.8%) | 2 (1.1%) | N/A |
| Any STH presence (n, %) | 388 (39.5%) | 311 (38.8%) | 77 (42.5%) | 0.159 |
| Hookworm presence (n, %) | 0 | 0 | 0 | N/A |
| *Ascaris lumbricoides* presence (n, %) | 329 (33.5%) | 264 (33.0%) | 65 (35.9%) | 0.237 |
| *Ascaris lumbricoides* EPG (mean ± SD) | 24.6 ± 58.8 | 21.7 ± 50.7 | 38.8 ± 87.0 | 0.114 |
| *Trichuris trichiura* presence (n, %) | 206 (21.0%) | 165 (20.6%) | 41 (22.6%) | 0.305 |
| *Trichuris trichiura* EPG (mean ± SD) | 2.2 ± 8.3 | 2.1 ± 8.5 | 2.9 ± 7.9 | 0.165 |
| *Loa* microfilaremia status (n, %) |  |  |  | 0.106 |
| Positive | 340 (34.6%) | 268 (33.5%) | 72 (39.8%) |  |
| Negative | 642 (65.4%) | 533 (66.4%) | 109 (60.2%) |  |
| *Loa* MFD (mfs/mL) |  |  |  | 0.062 |
| Mean ± SD | 2,425 ± 7,426 | 2,156 ± 6,829 | 3,613 ± 9,586 |  |
| Median [IQR] | 0 [0–540] | 0 [0–430] | 0 [0–1,460] |  |
| *Loa* MFD categories (n, %) |  |  |  | 0.087 |
| 0 mf/mL | 642 (65.4%) | 533 (66.5%) | 109 (60.2%) |  |
| 1–499 mfs/mL | 91 (9.3%) | 73 (9.1%) | 18 (9.9%) |  |
| 500–2,499 mfs/mL | 80 (8.1%) | 66 (8.2%) | 14 (7.7%) |  |
| 2,500–9,999 mfs/mL | 93 (9.5%) | 76 (9.5%) | 17 (9.4%) |  |
| ≥10,000 mfs/mL | 76 (7.7%) | 53 (6.6%) | 23 (12.7%) |  |

Abbreviations: PWV, pulse wave velocity; N, number; SD, Standard deviation; MFD, microfilarial density; IQR, interquartile range; N/A, not applicable.

* Chi-2 for categorical variable and Kruskal-Wallis rank test for continuous variables.

** An individual is defined as out of range if his/her PWV is higher than the 90^th^ percentile of the population considered healthy in the same age category (see S1 Table – References values #2)
