# Supplementary material for: Association between arterial stiffness and Loa loa microfilaremia in a rural area of the Republic of Congo: A population-based cross-sectional study (the MorLo project)
Source: PLoS Negl Trop Dis. 2024 Jan 19;18(1):e0011915. doi: 10.1371/journal.pntd.0011915 (PMC10830006; doi:10.1371/journal.pntd.0011915)
Supplement: S6 Table — Abbreviations: PWV, pulse wave velocity; bpm, beats per minute; aOR, adjusted odds-ratio; CI, confidence intervals. * An individual is defined as out of range if his/her PWV is higher than the 90th percentile of the population considered healthy in the same age category (see S1 Table–References values #2). (DOCX) [file pntd.0011915.s006.docx]

**S6 Table.** Results from logistic regression model explaining Pulse Wave Velocity status (using References values #2).

|  | PWV > 90^th^ percentile of the PWV in the healthy population (yes/no) * | | | | | |
| --- | --- | --- | --- | --- | --- | --- |
|  | aOR | 95% CI | p value | aOR | 95% CI | p value |
| Age | 0.98 | 0.97–0.99 | 0.001 | 0.98 | 0.97–0.99 | 0.001 |
| Sex |  |  |  |  |  |  |
| Female | Ref. |  |  | Ref. |  |  |
| Male | 1.11 | 0.73–1.70 | 0.600 | 1.10 | 0.72–1.68 | 0.659 |
| Smoking |  |  |  |  |  |  |
| No | Ref. |  |  |  |  |  |
| Yes | 0.91 | 0.58–1.44 | 0.695 | 0.91 | 0.57–1.43 | 0.672 |
| Average blood pressure |  |  |  |  |  |  |
| <100 mmHg | Ref. |  |  | Ref. |  |  |
| ≥100 mmHg | 2.10 | 1.44–3.06 | <0.001 | 2.07 | 1.42–3.02 | <0.001 |
| Pulse rate |  |  |  |  |  |  |
| <60 bpm | Ref. |  |  | Ref. |  |  |
| 60–90 bpm | 1.50 | 1.03–2.18 | 0.035 | 1.54 | 1.05–2.24 | 0.026 |
| >90 bpm | 1.67 | 0.65–4.29 | 0.286 | 1.76 | 0.68–4.54 | 0.242 |
| Body mass index |  |  |  |  |  |  |
| <18.5 kg/m^2^ | 1.08 | 0.67–1.74 | 0.737 | 1.10 | 0.68–1.77 | 0.703 |
| 18.5–25 kg/m^2^ | Ref. |  |  | Ref. |  |  |
| >25 kg/m^2^ | 1.11 | 0.63–2.95 | 0.713 | 1.12 | 0.64–1.99 | 0.679 |
| Creatininemia |  |  |  |  |  |  |
| <60 µmol/L | 0.62 | 0.39–0.99 | 0.044 | 0.60 | 0.37–0.96 | 0.032 |
| 60–110 µmol/L | Ref. |  |  | Ref. |  |  |
| >110 µmol/L | 1.65 | 0.65–4.19 | 0.287 | 1.64 | 0.65–4.17 | 0.294 |
| Any STH presence |  |  |  |  |  |  |
| No | Ref. |  |  | Ref. |  |  |
| Yes | 1.50 | 1.02 – 2.22 | 0.040 | 1.51 | 1.02 – 2.23 | 0.040 |
| MD | 1.57 | 1.01 – 2.45 | 0.047 | 1.56 | 0.99 – 2.44 | 0.053 |
| *Loa* microfilaremia status |  |  |  |  |  |  |
| Negative | Ref. |  |  |  |  |  |
| Positive | 1.38 | 0.98–1.95 | 0.068 |  |  |  |
| *Loa* MFD categories (mfs/mL) |  |  |  |  |  |  |
| 0 |  |  |  | Ref. |  |  |
| 1–499 |  |  |  | 1.17 | 0.66–2.09 | 0.582 |
| 500–2,499 |  |  |  | 1.12 | 0.60–2.10 | 0.719 |
| 2,500–9,999 |  |  |  | 1.16 | 0.64–2.10 | 0.609 |
| ≥10,000 |  |  |  | 2.36 | 1.35–4.11 | 0.002 |

**Abbreviations:** PWV, pulse wave velocity; bpm, beats per minute; aOR, adjusted odds-ratio; CI, confidence intervals

* An individual is defined as out of range if his/her PWV is higher than the 90^th^ percentile of the population considered healthy in the same age category (see S1 Table – References values #2)
