# Supplementary material for: Association between arterial stiffness and Loa loa microfilaremia in a rural area of the Republic of Congo: A population-based cross-sectional study (the MorLo project)
Source: PLoS Negl Trop Dis. 2024 Jan 19;18(1):e0011915. doi: 10.1371/journal.pntd.0011915 (PMC10830006; doi:10.1371/journal.pntd.0011915)
Supplement: Attachment — Submitted filename: Arterial stiffness and Loa loa microfilaremia Comments PLOSNTDs.docx [file pntd.0011915.s007.docx]

**Association between arterial stiffness and Loa loa microfilaremia in a rural area of the**

**Republic of Congo: a population-based cross-sectional study**

Jérémy T. Campillo et al examined is there any association between the density of *L. loa* microfilariae (mfs) in the blood with cardiovascular disease. They found that high *L. loa* MFD and cardiovascular health negative association which is deterioration.

Kindly mention about the consent details from study individuals under the ethical statement.

Study exclusion criteria’s should be included.

Sample size and the power calculation can be mentioned under statistical section.

Authors should include the methodology how they excluded the other helminth infection.

Flowchart for the study can be included

Did authors check for the post anthelminthic treatment effect on these cardiovascular markers. This data will improve the manuscript immensely

Did authors check for other confounding factors like Diabetes mellitus, lipid profile hypertension, etc…

Are these individuals are on treatment for cardiovascular disease? Do they have any impact on treatment?

Authors could show the comparison/ alteration of cardiac markers like NT-proBNP and high-sensitivity cardiac troponin T (hs-cTnT).

The above mentioned cardiac markers can be correlated with arterial stiffness and MFD

Discussion can be improved. Discussion regarding malaria and its impact is missing.

Study limitation and implication can be included at the end of the discussion.
