# Supplementary material for: Association between arterial stiffness and Loa loa microfilaremia in a rural area of the Republic of Congo: A population-based cross-sectional study (the MorLo project)
Source: PLoS Negl Trop Dis. 2024 Jan 19;18(1):e0011915. doi: 10.1371/journal.pntd.0011915 (PMC10830006; doi:10.1371/journal.pntd.0011915)
Supplement: Attachment — Submitted filename: Rev-PlosNTD - Loa - Stiffness - Responses .docx [file pntd.0011915.s008.docx]

**Dr. Subash Babu**

Academic Editor – PLOS Neglected Tropical Diseases

**Dr. Francesca Tamarozzi**

Section Editor – PLOS Neglected Tropical Diseases

Montpellier, November 30^th^ 2023.

**Manuscript PNTD-D-23-01213:** Association between arterial stiffness and *Loa loa* microfilaremia in a rural area of the Republic of Congo: a population-based cross-sectional study

Dear Drs. Babu and Tamarozzi,

Thank you for inviting us to revise and resubmit the above-mentioned manuscript. We have made the revisions according to the reviewers’ comments and are grateful for these thoughtful remarks. You will find below detailed comments about the way we handled each item in the revised version.

We have incorporated a flowchart to enhance the clarity of participant selection and the conducted analyses. Our discussion section has been expanded, incorporating the concepts recommended by the reviewers. Additionally, we have refined the English language throughout the manuscript for improved readability.

We are looking forward to your further thoughts on this manuscript along with those of the reviewers. All the authors have been involved with the revisions and agree with them.

Sincerely,

Dr Jérémy T. Campillo

**REVIEWER #1**

*In their manuscript, the authors report the results of a study examining arterial stiffness, ankle brachial index and pulse pressure in a large cohort of patients living in an area endemic for loiasis in an effort to explain the previously described association of microfilaremic loiasis with increased mortality. The objectives are clearly stated and the study design is appropriate.*

We thank reviewer 1 for his or her comments.

*Specific comments follow:*

*1 – A better description of the amicrofilaremic controls is needed. Were they infected (i.e., history of eye worm or Calabar swellings), uninfected, or both?*

Our analyses focused on microfilaremia alone as a marker of *Loa loa* infection. This decision was deliberate because we hypothesize that if cardiovascular complications exist in the context of loiasis, they are likely attributed to the presence of microfilariae in the vessels rather than the presence of adult worms which live in intermuscular fascia. Additionally, the region is highly endemic for loiasis. We added information about eyeworm episodes and Calabar swellings in the Methods section: “Among individuals with microfilaremia, 42.3% reported at least one eyeworm episode and 24.9% reported at least one episode of Calabar swelling during the previous year. Among amicrofilaremic individuals, 35.2% reported an eyeworm episode and 24.8% reported Calabar swellings” (lines 174-175).

We also add in the discussion the following sentence: “It should be noted that microfilaremic and amicrofilaremic subjects included in this study had similar frequencies of eyeworm episodes (42.3 vs. 35.2%, p=0.164) and of Calabar swellings (24.9 vs. 24.8%, p=0.961) during the last 12 months” (lines 461-463).

*2 – The description of the “reference populations” is unclear. Per the explanation in the methods, the difference between reference populations is only the definition of hypertension. As such, the assignment to normal or abnormal PWV group should be identical since this is an independent measure (i.e., why are there differences in line items other than arterial pressure?).*

To define our reference values for PWV analysis, we extracted a theoretically healthy population from our overall population. This included individuals who do not smoke, are not overweight, do not have hypertension, do not have known parasitic infections, and do not have lipid abnormalities or diabetes mellitus. From this sample population, we calculated the 90^th^ percentiles of PWV values by age and sex (as PWV varies with age and sex). Therefore, we defined our normal values as these 90th percentiles for each age/sex stratum. Each individual does not have the same 'normal value' (see Supplementary Material 1). Therefore, it is not solely an analysis focusing on arterial hypertension. This concept comes into play in defining our 'healthy population'. Hypertension has been defined as SBP ≥140 mmHg or DBP ≥90 mmHg for healthy population 1, and solely as the criterion of DBP ≥90 mmHg for healthy population 2.

For greater clarity, we have now included an illustrative figure (Figure 1) showing the construction of the reference population and the individuals classified as "out of range".

*3 – Although the authors state that the study was approved by the Congolese Ethics Committee, there is no mention of whether the participants provided informed consent.*

Certainly, participants provided their informed consent willingly to participate in the study. We added this information in the Ethics Statement part: “All participants received clear and appropriate information and signed an informed consent form for this specific study” (lines 152-153).

*4 – The relationship between Loa loa microfilaremia and PWV is not significant in Table 1 and demonstrated in Table 2 only for the highest mf load where >10,000 mf/mL is associated with a higher PWV. Although the Loa loa microfilaremia is associated with PAD, the way the Loa loa data is displayed in Table 4 is very difficult to follow and the logistic regression only showed a relationship in the low microfilaremia group. This seems at odds with the PWV data. The PP data, in contrast, would seem to support a relationship, but no data is given broken down by microfilarial load. In table 2 and supplementary table 6, there are two sets of data for each variable for each reference dataset. What is the difference between the two sets?*

Table 1 (bivariate results) and Table 2 (logistic regression results) for the PWV analysis utilize reference population #1, where the healthy population is characterized as non-smokers with no obesity, hypertension (**SBP ≥140 mmHg or DBP ≥90 mmHg**), parasitic infections, lipid abnormalities, or diabetes. Conversely, Tables S5 and S6 present the same analysis but use reference population #2, defining the healthy population as non-smokers with no obesity, hypertension (**DBP ≥90 mmHg**), parasitic infections, lipid abnormalities, or diabetes. To enhance clarity, we have included Figure 1. Furthermore, the information is provided beneath each table. For example, for Table 1, ** is: “An individual is defined as out of range if his/her PWV is higher than the 90^th^ percentile of the population considered healthy in the same age category (see Appendix p 1 – References values #1)”.

In relation to PP data, we opted to utilize microfilaremia solely in binary form rather than in categories. This decision was made to address convergence issues within the model.

*5 – Based on the findings (see comment in results section), the conclusion provided in the abstract that high Loa loa microfilarial load is related to cardiovascular deterioration seems overreaching.*

We appreciate the constructive feedback from reviewer 1. Taking their suggestions into consideration, we have tempered our abstract conclusion to better reflect our findings: "A potential link between high *L. loa* microfilaremia and cardiovascular health deterioration is suggested. Further studies are required to confirm and explore this association."

*6 – There are some additional confounders that should be addressed in the discussion.*

- *The authors point out that there no association between lipid levels and MFD. This does not exclude a role for the influence of genetic factors playing a shared role in the level of circulating microfilariae in an individual and their risk of cardiovascular disease.*

This is indeed a possible hypothesis, we thank the reviewer 1 for this comment. We added a sentence: “Nevertheless, the prospect of genetic factors influencing both the presence of circulating microfilariae and the risk of cardiovascular disease remains a possible avenue for future exploration” (lines 413-415).

- *Eosinophilia is common in loiasis, and eosinophil-platelet interactions and release of eosinophil extracellular traps have been reported to promote atherosclerosis (Marx et al. Blood 2019 among others). The potential role of eosinophils should be mentioned.*

We thank the reviewer for this very pertinent comment. We have made the requested addition to the discussion and have also added this reference. It is indeed possible that loiasis-induced eosinophilia plays a role in the cardiovascular complications we found: “A chronic inflammation, a mechanical effect of the mfs on the vasculature caused by high numbers of parasites passing through the vessels and/or loiasis-induced eosinophilia could induce such a phenomenon. Indeed, eosinophilia has been associated with atherosclerotic plaque formation and thrombosis.” (lines 454-456)

*7 – The public health relevance is not directly addressed and could be better highlighted in the discussion.*

We added a paragraph in the discussion about public health relevance of our findings: “Overall, the potential cardiovascular complications associated with loiasis may contribute to the elevated mortality observed in infected individuals. This phenomenon could emerge as a significant risk factor for cardiovascular mortality in the Central African context. Furthermore, it may exacerbate the escalating socio-economic challenges related to cardiovascular health in rural Africa, particularly given the limited availability of cardiologists and healthcare infrastructure for this population” (lines 469-474)

*8 – Although they bite horses, the common name for Chrysops is deerfly. Horsefly typically refers to the genus Tabanus.*

We thank the reviewer for this pertinent comment. We made the modification (line 107).

*9 – If p=0.073 is at “the borderline of significance” (line 263), why does p= 0.066 indicate a lack of significance (line 278). Since neither p value is <0.05, it would be more correct to indicate that neither is significant.*

Indeed, we agree with the reviewer. We made the changes: “*Loa loa* MFD (in binary, categories or continuous) was not significantly associated with out of range PWV measurements (respectively, p=0.107; p=0.073 and p=0.137)” lines 275-277).

*10 – On line 263, the authors state that the data in the supplemental table is similar to the data in Table 1; however, creatinine elevation is significantly different in the normal and abnormal PWV groups in the supplemental table.*

Indeed, we added this notion in the main text: “When the analysis used reference population #2 (hypertension defined as DBP ≥90 mmHg), the results were similar for *Loa loa* MFD but different for creatininemia levels with a significant elevation in the out of range PWV group” (lines 278-279).

*11 –There are many minor English errors that should be corrected.*

The manuscript has been fully proofread for English correction.

*12 – This is a novel study performed in a large cohort of patients in an area endemic for loiasis. Although the results are intriguing, the lack of sufficient information about the control population and lack of clarity in some of the data tables make it difficult to assess the overall significance of the results.*

We've added a new figure to make our analysis plan clearer. We have also modified our main text in line with reviewer 1's pertinent comments. We hope that our manuscript now meets the expectations of both the reviewer and the editor.

**REVIEWER #2**

*Jérémy T. Campillo et al examined is there any association between the density of L. loa microfilariae (mfs) in the blood with cardiovascular disease. They found that high L. loa MFD and cardiovascular health negative association which is deterioration.*

*1 – Kindly mention about the consent details from study individuals under the ethical statement.*

Participants provided their informed consent willingly to participate in the study. We added this information in the Ethics Statement part: “All participants received clear and appropriate information and signed an informed consent form for this specific study” (lines 118-119).

*2 – Study exclusion criteria’s should be included.*

As we wanted to represent the general adult population living in rural Congo, we had no exclusion criteria other than being an adult and having either no microfilaremia or at least 500 mf/mL in the previous 2019 screening. Figure 1 provides a better understanding of our sampling plan.

*3 – Sample size and the power calculation can be mentioned under statistical section.*

We appreciate the reviewer's insightful comment. It is important to clarify that no sample size was specifically calculated for this cross-sectional study; rather, the sample size determination pertained to the prospective MorLo cohort in the Congo, within which this study is nested. Recognizing the absence of preliminary data on arterial stiffness and loiasis, we opted to leverage the entire cohort population for this cross-sectional investigation. To enhance transparency and address concerns, we have incorporated a paragraph in the manuscript explaining our *a posteriori* power size calculation: “In this study, by employing a 1:2 matching design (1 microfilaremic matched with 2 amicrofilaremic individuals) and incorporating 990 participants, we anticipate a robust statistical power. Specifically, our design allows for nearly 100% power to detect a twofold higher risk (odds ratio, OR) of cardiovascular complications in microfilaremics compared to amicrofilaremics. Additionally, we expect a power of approximately 90% to identify a 1.5-fold higher risk” (lines 234-239).

*4 – Authors should include the methodology how they excluded the other helminth infection.*

For each patient, we looked for the presence of *Loa loa* microfilaremia, the presence of *Mansonella perstans* microfilaremia, the presence of antibodies against onchocerciasis, the presence of *Onchocerca volvulus* microfilaridermia in individuals with antibodies against the latter, the presence of malaria, and the presence of soil-transmitted helminth eggs in stools. In addition, the region is considered non-endemic for schistosomiasis. For the record, some individuals (those whose urine strip results showed an abnormality) also had an urinalysis, and no case of schistosomiasis was found.

Subjects with parasitic infections were therefore not excluded from the analyses, but included where possible (soil-transmitted helminths, and malaria, but in bivariate analysis only).

For clarity, we added the following sentence in the Methods section: “Despite schistosomiasis not being endemic in the area, any individual with haematuria underwent microscopic examination of urine (filtration method) for *Schistosoma* eggs and serological testing (LD BioDiagnostic®, *Schistosoma* ICT IgG IgM)” (lines 200-202) and in results “No schistosomiasis was found in the individuals with haematuria” (lines 270-271).

*5 – Flowchart for the study can be included*

We added the Figure 1 representing the flowchart of the study.

*6 – Did authors check for the post anthelminthic treatment effect on these cardiovascular markers. This data will improve the manuscript immensely*

We've added a sentence in the discussion on this limitation. Indeed, it would be unethical to treat hyper-microfilaremic patients without safe medication: “Moreover, at the time of the study, none of the patients reported taking medication for cardiovascular disease. Assessing the evolution of cardiovascular abnormalities after antihelminthic treatment could provide insights into the causality of lesions (if reversible) associated with *L. loa* microfilaremia, but there is currently no recommended treatment protocol in Congo for hyper-microfilaremia, and it would be ethically challenging to administer such treatment to these patients.” (lines 348-353)

*7 – Did authors check for other confounding factors like Diabetes mellitus, lipid profile hypertension, etc…*

We indeed checked for these confounding factors. All the results are available in supplementary materials Table 2 and 3 (lipid profiles and diabetes) and hypertension (average blood pressure) is used as an explanatory variable or as a definition of reference of PWV analysis.

*8 – Are these individuals are on treatment for cardiovascular disease? Do they have any impact on treatment?*

This comment is very pertinent. We thank the reviewer. None of the patients were taking treatment for cardiovascular disease at the time of the reviews. We have added a sentence mentioning this in the discussion: “Moreover, at the time of the study, none of the patients reported taking medication for cardiovascular disease” (lines 348-349).

*9 – Authors could show the comparison/ alteration of cardiac markers like NT-proBNP and high-sensitivity cardiac troponin T (hs-cTnT). The above mentioned cardiac markers can be correlated with arterial stiffness and MFD*

We thank the reviewer for this comment. While elevated values of PWV may suggest an increased cardiovascular risk, these values also actually signify an increased post-heart vascular resistance, thereby contributing to the negative effects on cardiac function and potentially leading to heart failure. This marker (NT-proBNP) is indeed useful for indirectly assess the impact on cardiac function. Unfortunately, due to the remoteness of our study region, conducting the assay under optimal conditions proved unfeasible. Moreover, cardiac ultrasounds were performed, and an in-depth analysis of cardiac function, along with its potential correlation with PWV, will be analyzed and reported in a dedicated article. This subsequent investigation will aim to examine the relationship between PWV and cardiac function, providing valuable insights into the cardiovascular implications associated with elevated PWV. Regarding hs-cTnT, this latter being mainly used for acute ischemic heart disease, we think that we cannot perform it at all our population study (as a systematic biological examination). In addition, kinetic disorders were more than rare (from cardiac ultrasounds; data not given in this paper); and we decided to examine post-ischemic lesions using solely cardiac ultrasounds.

*10 – Discussion can be improved. Discussion regarding malaria and its impact is missing.*

Several elements have been added to the discussion: the potential presence of genetic factors influencing both the presence of microfilaremia and the risk of cardiovascular, our results regarding malaria ("In this study population, less than 2% of the population exhibited malaria parasites in their blood, and no association with arterial stiffness has been shown," lines 417-420), the possibility of a mechanism induced by eosinophilia, and the impact on public health of our findings.

*11 – Study limitation and implication can be included at the end of the discussion.*

We modified the end of the discussion: “Certainly, the cross-sectional design falls and the lack of normative values for PWV analysis in our study population constrain the interpretability of our results. To address these limitations, further investigation is warranted to explore the long-term effects, including cardiovascular events and potential excess mortality, among the infected subjects included in this study, as well as to gain a deeper understanding of the mechanistic processes involved in ultrasound-based detection of mediacalcosis and/or atheroma. Overall, the potential cardiovascular complications associated with loiasis may contribute to the elevated mortality observed in infected individuals. This phenomenon could emerge as a significant risk factor for cardiovascular mortality in the Central African context. Furthermore, it may exacerbate the escalating socio-economic challenges related to cardiovascular health in rural Africa, particularly given the limited availability of cardiologists and healthcare infrastructure for this population.” (lines 428-438).
