# Supplementary material for: Association between arterial stiffness and Loa loa microfilaremia in a rural area of the Republic of Congo: A population-based cross-sectional study (the MorLo project)
Source: PLoS Negl Trop Dis. 2024 Jan 19;18(1):e0011915. doi: 10.1371/journal.pntd.0011915 (PMC10830006; doi:10.1371/journal.pntd.0011915)
Supplement: Attachment — Submitted filename: Rev2-PlosNTD - Loa - Stiffness - Responses .docx [file pntd.0011915.s009.docx]

**Dr. Subash Babu**

Academic Editor – PLOS Neglected Tropical Diseases

**Dr. Francesca Tamarozzi**

Section Editor – PLOS Neglected Tropical Diseases

Montpellier, January 9^th^ 2024.

**Manuscript PNTD-D-23-01213:** Association between arterial stiffness and *Loa loa* microfilaremia in a rural area of the Republic of Congo: a population-based cross-sectional study

Dear Drs. Babu and Tamarozzi,

Thank you for inviting us to revise and resubmit the above-mentioned manuscript. We have made the revisions according to the reviewers’ comments and are grateful for these thoughtful remarks. You will find below detailed comments about the way we handled each item in the revised version.

We are looking forward to your further thoughts on this manuscript along with those of the reviewers. All the authors have been involved with the revisions and agree with them.

Sincerely,

Dr Jérémy T. Campillo

**REVIEWER #1**

*Reviewer #1: The objectives are clearly stated and the study design is appropriate. Specific comments follow:*

*1)	Although Figure 1 helps in terms of clarifying the populations in the study, it is hard to follow and could be simplified. For example, out of range definitions and the definitions of the healthy cohorts (together with an explanation for why two separate definitions were used) would be better placed in the methods text describing the study populations.*

We appreciate your thorough examination of our manuscript and your insightful comments. Here are our responses to your specific points:

**Clarity on out of range definitions and healthy population:**

We acknowledge your suggestion regarding the relocation of out-of-range definitions and definitions of healthy cohorts from Figure 1 to the Methods section.

We would like to highlight that both out-of-range definitions and definitions of healthy cohorts are already presented in Figure 1 and in the Methods section. For instance, the out-of-range definition is detailed in Figure 1 and further elaborated in the Methods: “A PWV measure was considered ‘out of range’ for an individual if their PWV measure exceeded the 90th percentile of the constructed reference variable, for their specific sex and age category (Figure 1).[…]. PAD diagnosis was retained when ABI <0.90 [15]. PP was analyzed as a continuous variable.”; lines 169-177.

Similarly, the definition of healthy individuals is outlined both in Figure 1 and in the Methods section: “Using data from non-smoking individuals with no obesity/overweight (body mass index <25 kg/m2), no hypertension, no parasitic infection (STH, loiasis and malaria), no lipid abnormalities and no diabetes, 90th percentiles of PWV were calculated by sex and age categories (<30, 30–39, 40–49, 50–59, 60–69 and ≥70 years old) (Supplementary Material 1)” ; lines 165-169.

Our intent was to provide a comprehensive overview in Figure 1 while offering detailed explanations in the Methods section for those seeking more in-depth information. We believe retaining these details in Figure 1 enhances its comprehensiveness.

**Explanation for two separate definitions:**

We appreciate your observation regarding the use of two separate definitions for hypertension. We agree that this information is crucial. Two separate definitions were indeed used because hypertension includes different components. Initially, we employed the conventional definition, considering systolic blood pressure and diastolic blood pressure. Subsequently, we utilized an alternative criterion, where hypertension was identified solely based on a diastolic blood pressure. This second definition focuses specifically on peripheral resistance rather than cardiac flow, aiming to partially mitigate the 'white coat' effect often encountered during patient examinations.

This information is already available in the methods text (“Hypertension was defined in two ways: first, using the classic definition: SBP ≥140 mmHg or DBP ≥90 mmHg (Supplementary Material 1: References values #1); second, using only the criterion of DBP ≥90 mmHg (Supplementary Material 1: References values #2, Supplementary Material 5 and 6) focusing on peripheral resistance rather than cardiac flow to partially mitigate the "white coat" effect during patient examination”; lines 171-176).

*2)	Figure 1; 69 additional people were added. Were these microfilaremic, not microfilaremia or both?*

We have updated Figure 1 to specify the number of microfilaremic individuals in 2022 among the 69 new volunteers, as well as the number of microfilaremic individuals in 2019 among the 149 individuals not found. We hope these revisions adequately address your concerns.

*The authors have addressed most of the reviewers' comments; however, the two different sets of data in Table 2 and Supplementary Table 6 remain unexplained. Is the data in the left PWV >90th percentile "yes" and the data on the right "no"?*

Table 2 and Supplementary Table 6 each present two models. The first model, on the left, uses microfilaremia as a binary explanatory variable (presence or absence of microfilaremia). The second model, on the right, uses microfilaremia as a categorical variable (0; 1-499; 500-2,499; 2500-9,999; ≥10,000 mf/mL). The other explanatory variables are the same and the variable to explain is the same in each model (out of range PVW). We hope these explanations adequately address your concerns.
